# How many are affected? A real limit of epidemiology

**DOI:** 10.1186/1742-5573-7-6

**Published:** 2010-08-24

**Authors:** Charles Poole

**Affiliations:** 1 Department of Epidemiology, University of North Carolina, Chapel Hill, NC 27599-7435 USA

## Abstract

A person can experience an effect on the occurrence of an outcome in a defined follow-up period without experiencing an effect on the risk of that outcome over the same period. Sufficient causes are sometimes used to deepen potential-outcome explanations of this phenomenon. In doing so, care should be taken to avoid tipping the balance between simplification and realism too far toward simplification. Death and other competing risks should not be assumed away. The time scale should be explicit, with specific times for the occurrence of specified component causes and for the completion of each sufficient cause. Component causes that affect risk should occur no later than the start of the risk period. Sufficient causes should be allowed to have component causes in common. When individuals experience all components of two or more sufficient causes, the outcome must be recurrent. In addition to effects on rates and risks, effects on incidence time itself should be considered.

## Introduction

One hears much talk of epidemiology's limits [1-5], some real, some imagined. Gatto and Campbell [6] explore one of the real ones. Following previous attempts [7-9], they use Rothman's sufficient cause model [10] to explain how a person can experience a causative effect on the occurrence of an outcome in a defined follow-up period without experiencing an increase in the risk of that outcome over the same period.

Consider person GD3 in the authors' hypothetical example. She had the GSTm1 deletion and developed liver cancer at the end of the fourth year of a 12-year risk period. Had she lacked the deletion, she still would have developed liver cancer, but not until the end of the eighth year. The deletion therefore hastened her liver cancer by 4 years, unarguably a causative effect. Nevertheless, as the entire 4-year shift in her incidence time took place within the 12-year follow-up period, her 12-year risk was not affected. Neither was her 2-year risk. Her 6-year risk was increased, however.

Gatto and Campbell prefer to call a hastening of incidence time unaccompanied by an increase in risk "causal redundancy." The term is a fine one, except for its failure to convey the dependence of the phenomenon on an investigator's arbitrary choice of when to define the beginning and end of a risk period.

The basic idea, that an exposure contrast can hasten or delay the occurrence of a health outcome and that these causal effects may or may not be reflected as effects on risk over arbitrarily defined follow-up periods, is not very complicated. I usually find myself wondering if it needs to be explained in terms any deeper than those of potential outcomes. Many of us long for deeper explanations, however. When we do, we often turn to the model of sufficient causes.

In Gatto and Campbell's example, person GD3 had two potential liver cancer outcomes, one at the end of year 4 and the other at the end of year 8. She had two potential occurrences, each of which needs its own sufficient cause to explain it. The sufficient cause with the earlier completion time contained the GSTm1 deletion as a component cause. The one with the later completion time did not. The latter sufficient cause was a way of getting liver cancer to which the presence or absence of the GSTm1 deletion was irrelevant.

The success of Gatto and Campbell's example depends in part on how realistically the details of the sufficient causes and potential outcomes of person GD3 and the other members of the hypothetical cohort are portrayed. The example's success depends as well on the degree to which it brings aspects of the potential outcome and sufficient cause models, and their interface, into sharp relief.

## Assuming away a major competing risk

A remarkable feature of Gatto and Campbell's hypothetical example is that a cohort of 200 adults is followed for 12 years and no one dies. Death, of course, is a competing risk for liver cancer. When Robins and Greenland [11] developed the initial theory for the potential risk model employed here, it became considerably more complicated upon the introduction of competing risks. The assumption of more than a decade of temporary immortality in the present example is thus highly simplifying, but at the expense of working at cross purposes to the laudable goal of making the example realistic.

## Sufficient causes and risk

A sufficient cause is a list of conditions and events necessary for a given occurrence of a given outcome [10]. The items on the list are the component causes of which the sufficient cause is composed. A great plus of Gatto and Campbell's example is that the time scale, the occurrence time of each specified component cause and the completion time of each sufficient cause are all made explicit.

In the version of the sufficient cause model Gatto and Campbell employ, the occurrence of the final component cause is equivalent to the occurrence of the outcome [12-14]. The final component has an induction time of zero. When it acts, the outcome instantanously occurs. The outcome may not be instantaneously recognized, however. The latent period, or "the time it takes for the disease to become manifest" [10], is defined in this version of the model as the time between the occurrence of the outcome and its recognition by signs and symptoms.

This conceptualization differs from one in which the completion of a sufficient cause means not that the disease occurs, but only that "the disease process is set in motion" [10]. In this version of the model [9], time may elapse between sufficient cause completion and outcome occurrence. Here, the time it takes for a disease to "become manifest" [10] is not (or is not merely) the time between occurrence and recognition. This period includes (or is) the time it takes for the disease process that was set into motion by the completion of a sufficient cause to unfold and culminate, ultimately, in an occurrence of the disease.

When we specify a small number of components in a sufficient cause, we leave all the others unspecified. Some of the unspecified components may be known. The others are unknown. As a group, they form the "causal complement" [10] of the specified components.

A short-hand notation is usually used for the causal complement in each sufficient cause. In Gatto and Campbell's example, there are two sufficient causes of liver cancer. One, with a causal complement labeled U_1_, has the GSTm1 deletion and "aflatoxin acquisition" as the specified components. The other, with hepatitis C infection and "alcohol acquisition" as the specified components, has a causal complement labeled U_2_.

In studying effects on the risk of an outcome over a defined follow-up period, we properly relate the risk to each person's exposure history and status up to the start of that period (i.e., at baseline), but not beyond. In Gatto and Campbell's example, each cohort member's status with regard to the GSTm1 deletion was established long before baseline. The other specified component causes (hepatitis C infection and alcohol and aflatoxin "acquisition"), in contrast, take place years downstream of baseline. As it would not be possible to study the effects of these post-baseline exposures on the risk of liver cancer over the defined 12-year risk period, Gatto and Campbell make no attempt to do so.

Given that alcohol and aflatoxin "acquisition" are the final components of every sufficient cause in which they take part, we might imagine what it would take to study their effects on risk over any follow-up period. To study the aflatoxin effect, for instance, we could not be able to start each exposed person's risk period until she meets the operational definition of "acquiring" that exposure. Because this component cause is always the last one to complete a sufficient cause, its induction time is always zero in this version of the sufficient cause model. Hence, for every incident liver cancer the final component causes would occur at the very instant the follow-up period begins. As Gatto and Campbell assume uniformly zero latent periods in this version of the model, the disease would be instantly recognized as well.

The example could be modified to permit the study of the effects of alcohol and aflatoxin on risk by giving these component causes induction periods longer than zero. One then could roll back their "acquisition" to each person's status or history at the start of the 12-year follow-up period (i.e., at baseline). The last component cause to complete each sufficient cause could then be made an element of one of the causal complements, U_1 _or U_2_.

## Ubiquitous, uncorrelated causal complements

Gatto and Campbell follow Rothman and Greenland [13] and Rothman et al. [14] in constructing hypothetical examples in which all the causal complements are ubiquitous. In the present example, each person in the population is said to possess every single element of U_1 _and U_2_. The lack of realism in such assumptions has been acknowledged [14].

Gatto and Campbell go even further, however, in assuming that U_1 _and U_2 _share no component causes in common. It is not clear why this additional assumption was imposed. It would be unrealistic in many settings. Even when etiologic mechanisms for a given outcome differ in crucial respects, they often have at least some component causes in common. Some of the commonalities, such as the presence of tonsils for tonsillitis, are so obvious as to seem trivial. Trivial or not, they tend to create a positive "correlatedness of susceptibilities" [15] to the different sufficient causes. It would have done no harm that I can discern to have allowed U_1 _and U_2 _to share some elements in common in the present example.

## When an outcome is a competing risk for itself

Although Gatto and Campbell removed the major competing risk for liver cancer from their example by not letting anyone die in the 12-year follow-up period, they did allow one competing risk to remain. Following previous authors, they treated the outcome as a competing risk for its own subsequent occurrence (i.e., as a "nonrecurrent event") [13,14].

For recurrent outcomes such as diarrhea or myocardial infarction, it is relatively straightforward to depict a person who acquires every component of two or more sufficient causes. Had liver cancer been treated as such an outcome in the present example, person GD3 would have developed the disease twice: once at the end of year 4 when the sufficient cause involving aflatoxin, the GSTm1 deletion and U_1 _was completed and again at the end of year 8 upon completion of the sufficient cause involving alcohol, hepatitis C and U_2_.

Instead, Gatto and Campbell treated liver cancer as a non-recurrent event and, therefore, as a competing risk for itself. In this way, it was more akin to death or chicken pox than to diarrhea or myocardial infarction.

This decision created a quandary. Supposedly, all of the following were the case for person GD3: She had the GSTm1 deletion at the start of the risk period. Possessing every element of U_1 _as well, she developed liver cancer at the end of year 4 when she "acquired aflatoxin." Meanwhile, she had become infected with hepatitis C at the end of year 2. After her first liver cancer, and in possession of every element of U_2_, she went on to drink enough alcohol over the next 4 years to "acquire" the final component of a second sufficient cause. *But she did not develop a second liver cancer at that time*. Somehow, she experienced every single component of a sufficient cause of an outcome (an incident liver cancer at the end of year 8) without experiencing that outcome. This cannot be.

From the fact that person GD3 experienced liver cancer at the end of year 4, she must have experienced every element of U_1 _in addition to having the GSTm1 deletion and "acquiring" aflatoxin. From the fact that she did *not *experience a second liver cancer at the end of year 8, despite developing a hepatitis C infection and "acquiring alcohol," I believe we must conclude that she did not experience every element of U_2_. Thus, all the elements of U_2 _were not ubiquitous after all. In fact, given the portrayal of liver cancer in this example as a competing risk for itself, it would seem that at least one element of U_2 _must have been the negation of (or the well-specified alternative to) at least one element of U_1_.

The example is similar in this regard to an example constructed by Rothman et al. [14] with three sufficient causes. One contained A = 0, B = 1 and U_1_. The second contained A = 0, E = 1 and U_2_. The third contained B = 1, E = 1 and U_3_. The three causal complements (U_1_, U_2 _and U_3_) were said to be ubiquitous. Each individual with the exposure pattern A = 0, B = 1, E = 1 should have experienced the outcome 3 times, once for each of the completed sufficient causes she experienced. But the outcome was said to be non-recurrent, so each of these individuals experienced the outcome only once. Hence, the example contained the same logical contradiction as in Gatto and Campbell's example: ostensibly completed sufficient causes that do not culminate in occurrences of the outcome.

I would suggest that it is not possible under the terms of this version of the sufficient cause model for an individual to experience all the specified components of 2 or more sufficient causes with different completion times, for the causal complements of those sufficient causes to be ubiquitous, and for the outcome to be non-recurrent. If a person experiences all the specified and unspecified components of 2 or more sufficient causes with different completion times, that person must experience the outcome more than once. If a person experiences all the specified components of 2 or more sufficient causes and the outcome is non-recurrent, each causal complement must contain at least one component that is the negation of (or the well-specified alternative to) a component of each of the other sufficient causes and, therefore, the causal complements cannot be ubiquitous.

## Rates and risks are not the only options

Students and other collaborators sometimes tell me they do not want to study risk. Instead, they want to study time to event. I congratulate them and ask how we are going to go about that task. The usual answer is that we are going to fit a proportional hazards model. When I ask what that analysis will tell us about time to event, my collaborators seem befuddled. So I ask why we are going to fit a proportional hazards model. The answer tends to be something like this: "Because it will give us something like a relative risk." These conversations make me wonder how strong the initial commitment to studying time to event really was.

Gatto and Campbell consider an analysis of incidence rates, but not an analysis of incidence times [16]. In their hypothetically perfect study of temporarily immortal persons, the causal effect of the GSTm1 deletion in the 100 members of the study population who had that deletion (i.e., in the authors' chosen target population) was to reduce the liver cancer-free survival time from 1,187 to 1,167 person-years at risk. Averaged over the entire target population, the total reduction of 20 years would amount to a reduction of 0.2 years, or 2.4 months per person.

If by "the full etiologic effect" the authors mean the number of individuals in the target population who had their liver cancer experience causally affected in any way over the 12-year follow-up period, I suspect they would be just as disappointed with this analysis as they were with the analyses of risks and rates. No epidemiologic data analysis would be capable of showing the number of individuals affected, even in this hypothetically perfect study with the highly simplifying assumptions of no competing risk from death and no ability of the GSTm1 deletion to prevent or delay any occurrence of liver cancer. It could have been that all 5 cases had their incidence times hastened by an average of 4 years. Or the effect could have been experienced by 4 of the 5 cases, for an average reduction of 5 years. Or the entire causative effect on liver cancer-free survival time could have been confined to the three excess cases, for an average of reduction of 6.7 years.

The first of these scenarios was the correct one, but we know that only because we know each person's potential incidence times. If we knew such things in real life, we would not need to do epidemiologic research.

## Conclusion

On the first page of the first epidemiology textbook [17], Greenwood wrote:

The physician's unit of study is a single human being, the epidemiologist's unit is not a single human being but an aggregate of human beings, and since it is impossible to hold in the mind distinctly a mass of separate particulars he forms a general picture, an average of what is happening, and works upon that.

Because we work with averages, we will almost never know, without very strong assumptions, how many individuals are affected by the conditions and events we study. The mass of separate particulars of individual effects cannot be held distinctly in our minds, or in our methods.
